# Heterotopic Pregnancy: Case Series and Review of Diagnosis and Management

**DOI:** 10.1155/2023/2124191

**Published:** 2023-05-05

**Authors:** Somaia Elsayed, Nadine Farah, Mary Anglim

**Affiliations:** The Coombe Women and Infants University Hospital, Dublin, Ireland

## Abstract

**Introduction:**

Heterotopic pregnancy (HP) refers to the simultaneous presence of intrauterine pregnancy (IUP) and ectopic pregnancy, which is very rare but potentially life-threatening. The spontaneous incidence of HP in the general population is 1/30,000. With the widespread use of assisted reproductive technology (ART), the incidence rises to 1/1,000. *Aims and Methods.* This is a prospective case series looking at the cases of heterotopic pregnancies presenting to the early pregnancy unit (EPU) in a tertiary maternity hospital, from November 2015 to November 2016. The clinical presentation, ultrasound findings, and laparoscopy findings were all documented. The incidence of HP was calculated and compared with the quoted incidence in the literature. *Outcomes.* Five women with HP presented to the EPU over the course of a year. The first case describes a spontaneous HP with a previous salpingostomy. The second case describes an HP following ovulation induction. The third case describes a spontaneous HP with no known risk factors. The fourth and fifth cases describe heterotopic pregnancies following in vitro fertilisation with more than one embryo. All five cases of HP underwent laparoscopy and salpingectomy with uneventful recovery. The three women who had a viable IUP had no further complications in their pregnancies.

**Conclusion:**

Early and accurate diagnosis of HP can be challenging. An early transvaginal ultrasound plays an important role in making the diagnosis in women with risk factors and following ART. A high index of suspicion is required for timely diagnosis and appropriate intervention, especially in spontaneous HP.

## 1. Introduction

A heterotopic pregnancy (HP) is an intrauterine pregnancy (IUP) and an ectopic pregnancy (EP) that occur simultaneously. It was first reported in 1708 as an autopsy finding [1]. It is a rare condition that can be potentially life-threatening and can be either spontaneous or the consequence of assisted reproductive technology (ART) [2]. The incidence of HP in spontaneous conceptions is reported to be around 1 in 30,000 pregnancies [3]. In women who have conceived with the use of ART, the incidence of HP rises to 1 in 1,000 [3, 4]. However, clinicians often overlook this diagnosis in cases presenting with abdominal pain or vaginal bleeding, particularly when an IUP has already been identified.

As with all ectopic pregnancies, the EP part of an HP occurs most commonly in the fallopian tube. However, other types like ovarian and cervical HP have also been reported [5, 6]. Most heterotopic pregnancies are singleton intrauterine pregnancies, but triplet and quadruplet heterotopic pregnancies have also been reported. These are extremely rare and occurred mostly following assisted reproduction [7, 8].

Risk factors for HP are similar to those for EP and include tubal damage, which can be a consequence of pelvic inflammatory disease, endometriosis, or previous tubal surgery [2, 9]. Other major risk factors are ovulation induction and other forms of assisted reproduction [2].

HP can be a challenging diagnosis. In up to 50% of cases, the woman is initially asymptomatic [2, 3, 10, 11]. When symptoms do occur, they are variable and include abdominal pain and vaginal bleeding. These symptoms are often observed in an IUP or threatened miscarriage. Therefore, ultrasound plays a key role in making the diagnosis [11], but the diagnosis can still sometimes be missed or overlooked due to the presence of the IUP [5]. Furthermore, serum beta-human chorionic gonadotrophin (*β*-hCG) levels are not helpful in the diagnosis of HP, particularly in the presence of a viable IUP [2]. Therefore, HP may be discovered late, increasing the risk of considerable life-threatening haemorrhage and hypovolemia as a consequence of the EP rupturing [12, 13].

## 2. Materials and Methods

This was a prospective case series looking at heterotopic pregnancies presenting to the early pregnancy unit (EPU) at a tertiary maternity hospital, from November 2015 to November 2016. The clinical presentation, ultrasound images, and outcomes were documented. The incidence of HP in our hospital was calculated and compared with the incidence quoted in the literature.

## 3. Results

Five women presented to the EPU with HP over the course of one year.

### 3.1. Case 1: Spontaneous HP with a Previous EP

An asymptomatic 33-year-old, gravida 4, para 2 (two uncomplicated term pregnancies and one tubal EP with laparoscopic right salpingostomy), presented at seven week's gestation for an early pregnancy ultrasound given a previous history of an EP.

Transvaginal ultrasound revealed a viable IUP with a 6.2 mm fetal pole. On examination of the adnexa, a right-sided viable EP was noted with a gestational sac and a fetal pole. No free fluid was noted.

**Figure 1:**
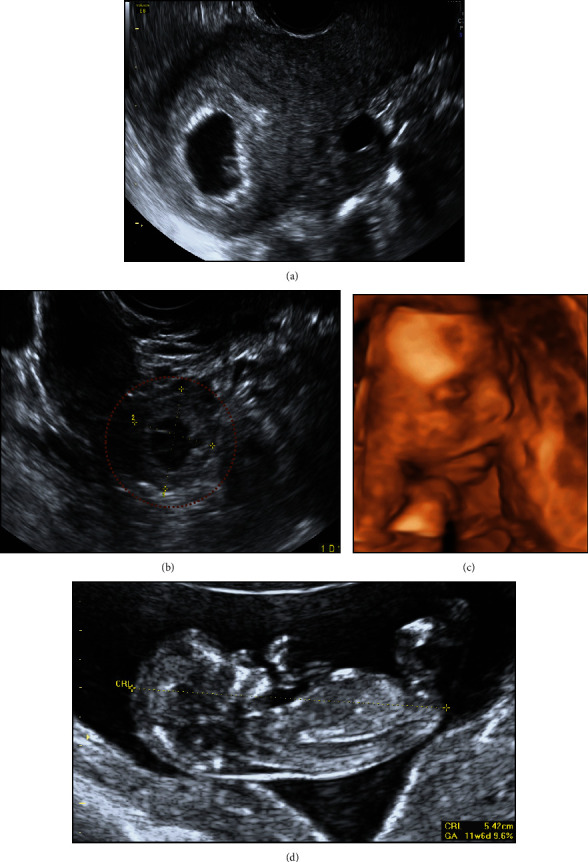
Case 1: spontaneous HP with a previous EP. (a) IUP. (b) Right-sided EP. (c) Normal dating scan. (d) 3D scan at 20 weeks.

She underwent laparoscopic right salpingectomy. Post-operatively, a viable IUP was confirmed on ultrasound. She had no further complications in this pregnancy and had a spontaneous vaginal delivery of a baby boy (3.6 kg) at 40 + 4 weeks' gestation (Figure 1).

### 3.2. Case 2: HP following Assisted Reproduction

**Figure 2:**
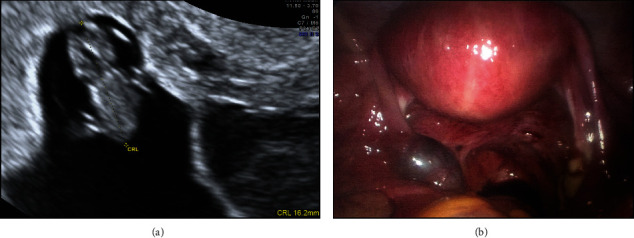
Case 2: HP following assisted reproduction. (a) IUP. (b) Left-sided tubal EP seen at laparoscopy.

A 37-year-old, gravida 8, para 1 (six spontaneous miscarriages and one term pregnancy) underwent ovulation induction. She was referred to our unit for management following an ultrasound in the fertility unit at 7 + 5 weeks gestation, which identified a HP with a left-sided tubal EP. She underwent laparoscopic left salpingectomy. A transvaginal ultrasound the following day confirmed an ongoing IUP with a 16 mm fetal pole (Figure 2). The IUP was uncomplicated, and she had a spontaneous vaginal delivery of a baby girl at 40 weeks gestation.

### 3.3. Case 3: Spontaneous HP with No Risk Factors

A 38-year-old, gravida 2, para 1 (one full-term pregnancy) presented to the emergency room with a two-day history of left iliac fossa (LIF) pain and minimal vaginal bleeding. LIF tenderness was noted on examination, but no cervical motion tenderness was elicited. A transvaginal ultrasound revealed an IUP with a mid-gestational sac diameter of 12.7 mm, and a yolk sac, but no fetal pole. A 20 mm × 20 mm right ovarian haemorrhagic cyst and a left ovarian simple cyst of 24 mm × 21 mm were also noted. No free fluid was present. A diagnosis of pregnancy of uncertain viability was made. A follow-up scan was organised 14 days later to determine viability.

**Figure 3:**
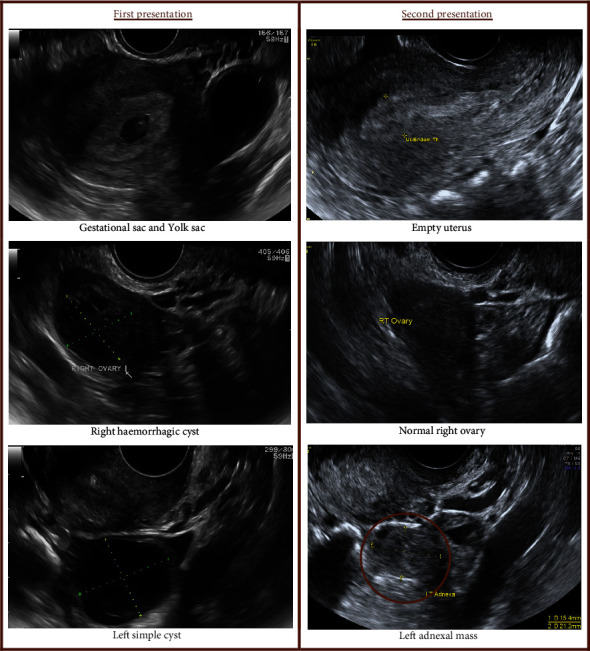
Case 3: spontaneous HP with no risk factors.

She presented again to the emergency room 12 days following discharge with severe LIF pain and heavier vaginal bleeding. On transvaginal ultrasound, the intrauterine gestational sac was not visible anymore, and the endometrium was disrupted and measured 13 mm. Both ovarian cysts noted on the last scan had resolved. There was a small amount of free fluid in the pouch of Douglas. A left adnexal heterogeneous mass of 15 mm × 21 mm was noted. A diagnosis of an incomplete miscarriage, a ruptured ovarian cyst, and a suspected left EP was made (Figure 3). She underwent laparoscopic left salpingectomy and a surgical evacuation of retained products of conception from the uterus. The histopathology confirmed the presence of both pregnancies.

### 3.4. Case 4: HP following Assisted Reproduction with a Miscarriage and a Delayed Diagnosis of EP

**Figure 4:**
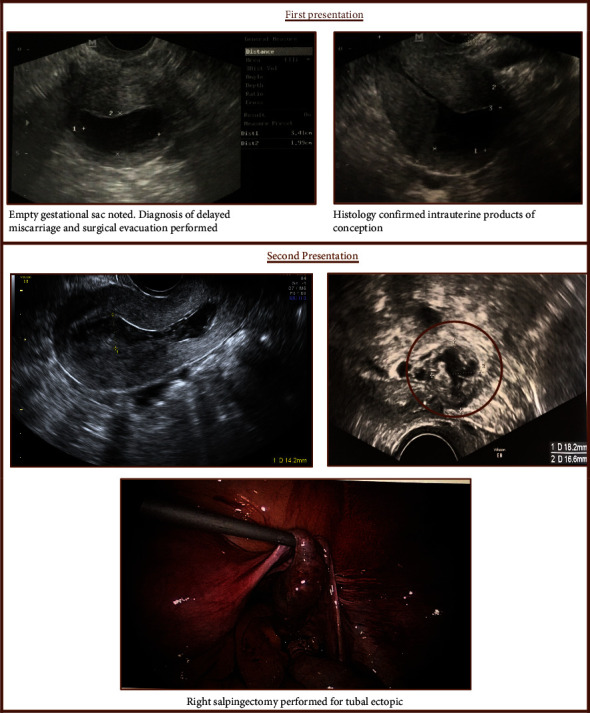
Case 4: HP following assisted reproduction with a miscarriage and a delayed diagnosis of EP.

A 45-year-old underwent a two-embryo transfer and was referred following a diagnosis of a non-viable pregnancy at 9 weeks gestation. Transvaginal ultrasound demonstrated an empty gestational sac of 48 mm. She underwent surgical evacuation of the uterus three days later and was discharged home. She presented 12 days later with heavy vaginal bleeding. A transvaginal ultrasound revealed an enlarged uterus with a blood-filled uterine cavity. She was diagnosed with an incomplete miscarriage and possible endometritis. However, following a serum hCG of 504 mIU/ml, she underwent another ultrasound. A right adnexal mass of 18 mm was noted. She underwent laparoscopic right salpingectomy for a right tubal ectopic and repeat uterine evacuation (Figure 4). Histopathology confirmed the presence of tubal ectopic and intrauterine products of conception.

### 3.5. Case 5: HP following Assisted Reproduction and a History of an EP

**Figure 5:**
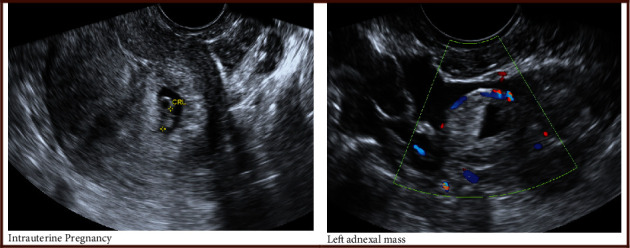
Case 5: HP following assisted reproduction and a history of an EP.

A 41-year-old, gravida 4, para 1 (one miscarriage following assisted reproduction, one right EP with no surgery, and one uncomplicated pregnancy), underwent a two-embryo transfer. She presented at seven weeks' gestation with abdominal pain. A transvaginal ultrasound identified a viable IUP with a 20 mm left adnexal mass. She underwent laparoscopic left salpingectomy for a left tubal EP. Repeat ultrasound after surgery confirmed an ongoing IUP Figure 5). She had an uncomplicated delivery at term.

## 4. Discussion and Review of HP

HP is a rare condition that has increased in incidence with ovulation induction and assisted reproduction. However, clinicians often overlook this diagnosis in cases presenting with abdominal pain or vaginal bleeding, particularly when an IUP has already been identified.

The overall incidence of HP in our hospital over the period that this case series was written was 0.05% (1 in 2,000). The incidence was calculated using the number of all pregnancies including all miscarriages in this unit: 10,098 pregnancies. Our incidence of spontaneous HP was 0.02% (1 in 5,000, excluding the cases following assisted reproduction). This incidence appears to be higher than the quoted figures in the literature (1 in 15,000/30,000) [14]. One factor contributing to this higher rate could be improved ultrasound technology identifying some of the heterotopic pregnancies that would have resolved spontaneously. Increased maternal age in our population and increased rate of ART may also have been a factor as this contributes to the increased rate of multiple pregnancies, some of which can be extrauterine. Furthermore, the improved outcomes of EP treatment result in more women with risk factors getting pregnant again.

EP continues to be a significant cause of maternal morbidity and mortality in the first trimester of pregnancy. Understanding the factors that increase the risk of EP is essential to improving pregnancy outcomes as well as preserving future fertility [13]. Risk factors for HP are similar to EP. Women with an HP are at an increased risk of a spontaneous or medically induced miscarriage and are 30% less likely to have a live-birth delivery compared with women with isolated intrauterine pregnancies [15].

The risk factors for EP and HP are many and include pelvic inflammatory disease, previous ectopic pregnancies, previous tubal surgery, endometriosis, the use of intrauterine devices, maternal age over 35 years, smoking, and infertility treated with ART [14]. In four of the cases in this study, the women had risk factors for HP (previous tubal surgery for EP and ovulation induction). In a review of HP from 1994 to 2004 by Barrenetxea et al., 63% of 80 cases of HP had a history of previous tubal surgery, 35% had a history of previous EP, whereas 16% of HP were spontaneous with no risk factors [2].

The greatest risk factor for the development of HP is ART. The risk of extrauterine pregnancy is up to eight times greater in women undergoing assisted reproduction compared with the background risk of EP [16]. Different mechanisms may predispose toward the development of HP after assisted reproduction. Ovulation induction and the transfer of more than one embryo increase both the multiple gestations and EP rates [17]. Another factor predisposing to ectopic gestation is tubal damage or previous tubal surgery, which is an important indication for undergoing ART [2, 17]. Furthermore, research has shown that frozen or donor cycles without ovarian stimulation were associated with lower rates of EP compared with fresh autologous cycles. In a study by Londra et al., a paired analysis among 908 women who underwent both a fresh and a frozen-thawed embryo transfer with autologous oocytes on separate occasions found that odds of EP were lower with frozen-thawed transfer than with fresh transfer (OR = 0.22, 95% CI 0.16–0.31; *P* < 0.001) [13]. These factors are important to consider when assessing EP and HP risk factors in women who conceived through ART.

Early diagnosis of HP is challenging, especially when the patient is asymptomatic. The signs and symptoms of a HP are subtle and often absent on first visits, which can lead to a delay in diagnosis. Transvaginal ultrasound is key to making the diagnosis [12, 16]. The ultrasound visualization of fetal heart activity in both the intrauterine and the extrauterine gestations is important for confirming the diagnosis, but its presence is rare [18]. An HP can be misdiagnosed on ultrasound examination as a luteal cyst, especially if the concurrent IUP is reassuring [19]. On the other hand, intrauterine gestation with hemorrhagic corpus luteum can simulate heterotopic/ectopic gestation both clinically and on ultrasound. [20]. Furthermore, the presence of ovarian pathology, such as cysts, can mask other adnexal masses and make the visualization of an EP technically difficult. This difficulty can also be an issue following ovulation induction or in the presence of ovarian hyperstimulation syndrome where the ovaries can be enlarged and multi-cystic [2]. Other surgical conditions causing an acute abdomen can also simulate heterotopic gestation clinically [21]. In a study by Jeon et al., despite the early transvaginal ultrasonography performed in patients with IVF, only 16% of asymptomatic heterotopic pregnancies were diagnosed, concluding that early diagnosis is difficult [12]. Offering routine early pregnancy ultrasound can be critical in the early diagnosis of patients with known risk factors and can improve the outcomes [22]. Presenting early in the pregnancy could prevent the rupture of the EP and the subsequent morbidity.

A HP significantly differs from a normal extrauterine pregnancy due to the importance of preserving the IUP. For this reason, possible treatments, such as methotrexate, are contraindicated. This makes the treatment of HP difficult and challenging [1, 22, 23]. Treatment modalities of HP are expectant management, surgical management, and sonographic-guided embryo aspiration with or without embryo-toxic drugs [1, 11, 22, 24]. Due to the rarity of HP, most publications about HP are single case reports or small case series, with no consensus on the preferred treatment modality of HP [25]. The treatment options depend on the location of the EP and whether the IUP is viable or not. However, surgical intervention has long been the ‘gold standard' for the treatment of EP [26].

In hemodynamically stable and asymptomatic women, expectant management could be considered [6, 23, 27]. The main advantage of expectant management is avoiding all potential complications related to the surgical management [6, 26]. However, expectant management should not be considered in patients with a viable or large EP due to the high risk of rupture of the EP [28]. In a study by Li et al., 20% of patients undergoing expectant management suffered a rupture of the EP [25]. Regular ultrasonographic examination with immediate access to surgical management is essential in women undergoing expectant management. Follow-up with serum hCG is not useful, particularly in the presence of a viable IUP as the level of hCG will continue to rise [2].

Surgical management by laparoscopic salpingectomy in a tubal EP is generally a safe procedure with no increase in fetal loss rate in the IUP [12]. Surgical management has the advantage of complete removal of the EP mass. However, the outcomes and the complexity of the surgery can vary depending on the location of the ectopic component of the HP. In a study by Jeon et al., a live birth rate of 80% in HP was reported. The live birth rate was higher in tubal HP compared with the interstitial HP [12]. In the case of a tubal EP, various techniques are possible including salpingectomy, salpingostomy, or less commonly tubal anastomosis. Recent research suggests similar subsequent fertility rates following the different surgical approaches. A cohort study by Li et al. showed the 2-year cumulative recurrent EP rates were found to be 8.1% for salpingectomy, 6.3% for salpingostomy, and 16.7% for tubal anastomosis treatments [26]. Patients with an EP receiving tubal anastomosis treatments appeared to have a lower 2-year rate of IUP and a higher risk of recurrent EP after adjustment for other potential risk factors. Persistent trophoblast occurred significantly more often in the salpingostomy (9.8%) and tubal anastomosis (8.3%) group than in the salpingectomy group (1.8%). Given this data, salpingectomy is probably the preferred approach particularly if the other fallopian tube appears normal [26].

Transabdominal sonographic guided aspiration of the ectopic gestation, with or without embryo-toxic drug, is another treatment modality of the EP component in an HP. Its safety and effectiveness have been well demonstrated [4, 29]. It is minimally invasive, but can be technically difficult depending on the location of the ectopic gestational sac. It should be attempted only when the ectopic gestational sac is clearly visualized [2]. Both potassium chloride and hyperosmolar glucose can be used as embryo-toxic drugs in the management of HP, whereas methotrexate should be avoided because of its teratogenic effects on the viable intrauterine-pregnancy [27].

The chosen treatment approach for the ectopic component of HP can depend on multiple factors. These include the type of EP, whether the IUP is viable or not, how symptomatic the pregnant woman, and the expertise of the treating clinician.

## 5. Conclusion

An early pregnancy transvaginal ultrasound scan should be offered to women at high risk for EP. When performing an ultrasound in early pregnancy, HP should be suspected in patients with an adnexal mass, even in the absence of risk factors. Clinicians must be alert to the fact that confirming an IUP clinically or by ultrasound does not exclude the coexistence of an EP. A high index of suspicion in women is needed for early and timely diagnosis, and management with laparoscopy can result in a favourable and successful obstetrical outcome.

## Data Availability

Data supporting this research article are available from the corresponding author or first author on reasonable request.
